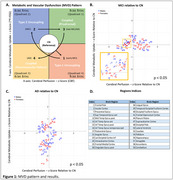# Metabolic and Vascular Dysfunction of Brain Regions in Alzheimer's Disease and Related Dementia

**DOI:** 10.1002/alz70862_110124

**Published:** 2025-12-23

**Authors:** Juan Antonio Kim Hoo Chong Chie, Scott A Persohn, Ravi S Pandey, Olivia Simcox, Paul Salama, Paul R Territo

**Affiliations:** ^1^ Stark Neuroscience Research Institute, Indiana University School of Medicine, Indianapolis, IN USA; ^2^ Stark Neurosciences Research Institute, Indiana University School of Medicine, Indianapolis, IN USA; ^3^ The Jackson Laboratory, Bar Harbor, ME USA; ^4^ Marian University, Indianapolis, IN USA; ^5^ Purdue School of Engineering and Technology, Indianapolis, IN USA; ^6^ Indiana University School of Medicine, Indianapolis, IN USA

## Abstract

**Background:**

Historically, clinical diagnosis of ADRD via medical imaging has largely focused on singular modalities late in the disease process to identify structural and/or functional changes; however, by this point, irreversible brain damage has occurred, thus resulting in a lack of sensitivity and specificity of the measure. Since treatment planning is often based on these readouts, outcomes are often compromised, leading to ineffective treatment responses. Recent preclinical data suggests that regional metabolic and vascular dysfunction (MVD) persist across the disease spectrum; however, few studies have explored these combined measures in ADRD. Therefore, we hypothesized that MVD may serve as a sensitive and early noninvasive diagnostic tool, and tested this in a retrospective clinical population spanning from CN to AD.

**Method:**

Subjects from ADNI who were scanned with ^18^F‐FDG PET and ASL MRI within 180 days were included (*N* = 290; CN(91), MCI(116), AD(83), M/F(51.2/48.8%)). Cerebral blood flood (CBF) and images were generated from ASL images using ExploreASL. PET and CBF images were registered to the augmented MNI152+ Atlas. Mean intensities for unilateral 59 brain regions were ratioed to whole brain values, *z*‐scores computed for each region (relative to CN), and were projected into Cartesian space. Statistical analysis of at‐risk regions was evaluated using Student’s *t*‐test (*p* <0.05), and hierarchical clustering of at‐risk regions was aligned with transcriptomic signatures and clinical cognitive assessments.

**Result:**

Our findings suggest that disease progression follows an MVD pattern (Figure 1A), consistent with our preclinical work, illustrating the suitability of the approach for stage‐dependent detection of at‐risk brain regions. Moreover, clustered regions associated with memory, cognitive, and motor functions progress at different rates, where MCI subjects show both Type 1 (↓^18^F‐FDG,↑CBF) and Type 2 (↑^18^F‐FDG,↓CBF) uncoupling MVD (Figure 1B); where AD subjects showed prodromal (↑^18^F‐FDG,↑CBF) and neuro‐metabolic‐vascular failure (↓^18^F‐FDG,↓CBF) MVD phenotype (Figure 1C). Importantly, these changes aligned with transcriptomic and cognitive signatures.

**Conclusion:**

Our findings show that MVD of the at‐risk brain regions depends on sex, APOE status, age, and disease stage. Moreover, our data reveals that MVD patterns can provide a sensitive diagnostic tool for early diagnosis of ADRD, which may improve patient monitoring, stratification, and therapeutic testing.